# Image-Based Fractional Flow Reserve: Art and Science. Reply to Taylor et al. Single View Techniques for Modelling Coronary Pressures Losses. Comment on “Tsigkas et al. Rapid and Precise Computation of Fractional Flow Reserve from Routine Two-Dimensional Coronary Angiograms Based on Fluid Mechanics: The Pilot FFR2D Study. *J. Clin. Med.* 2024, *13*, 3831”

**DOI:** 10.3390/jcm14062086

**Published:** 2025-03-19

**Authors:** Grigorios G. Tsigkas, George C. Bourantas, Athanasios Moulias, Grigorios V. Karamasis, Fivos V. Bekiris, Periklis Davlouros, Konstantinos Katsanos

**Affiliations:** 1 Department of Cardiology, University Hospital of Patras, 26504 Patras, Greece; dramoulias@live.com (A.M.); pdav@upatras.gr (P.D.); 2 Medlytic Labs, 26222 Patras, Greece; georgios.bourantas@gmail.com (G.C.B.); fbekiris@gmail.com (F.V.B.); katsanos@med.upatras.gr (K.K.); 3 Second Cardiology Department, Attikon University Hospital, National and Kapodistrian University of Athens Medical School, Rimini 1, Chaidari, 12462 Athens, Greece; grigoris.karamasis@gmail.com; 4 Department of Interventional Radiology, School of Medicine, University of Patras, 26222 Patras, Greece

We read the response of Taylor et al. to our “pilot FFR2D study” with great interest and we take the opportunity to expand and discuss further some important preconditions [1], modelling assumptions, and associated challenges of the latest image-based software devices for the calculation of Fractional Flow Reserve (FFR) in the coronary catheterization laboratory. Current methods of calculation of angiographic FFR—also known as ‘virtual’ FFR—broadly include analytical mathematical models, like our FFR2D approach or the QFR and μFR techniques by Tu et al. [2,3], or high-fidelity Navier–Stokes computational fluid dynamics, like in the competing cases of the vFFR proposed by Morris et al. [4,5], or even the Heartflow Inc. approach in case of coronary computed tomography angiography (CCTA) acquisitions [6]. Regardless, it is very much true that the accuracy and precision of the end-result—i.e., predicted FFR values—depend heavily on a complex interplay between the anatomical input (details of the vascular geometry under study) and the physiological conditions (simulated hyperemic flow and microvascular resistance), and are often driven more by statistical modeling than absolute scientific principles.

Most virtual FFR systems rely on the reconstruction of a 3-dimensional anatomy from two (VirtuHeart, QFR, vFFR, etc.) or even three different angiographic runs (FFRAngio by Cathworks) by epipolar geometrical techniques. However, with the rare exception of biplane imaging, this mandates more than one angiographic run, increasing patient contrast dose and radiation exposure. Unfortunately, the system often suffers from registration errors or even failure to reconstruct the 3-dimensional anatomy. Study-reported rates of non-analyzable angiograms vary from 7% to as much as 18% [7,8]. Moreover, CFD models may suffer from further failure to produce a suitable (high-quality) mesh or convergence of the solver. On the contrary, single-view angiographic analyses are inherently simpler, faster, readily available, and more successful in computing virtual FFR. We reported a 3.3% rate of retrospectively non-analyzable cases for the pilot FFR2D study; we postulated that this may be well <1.0% in prospective real-world applications, assuming good coronary angiographic practice is involved [9].

Arguments favoring the need for a 3-dimensional geometry include better representation of the geometrical characteristics of the stenosis—such as its severity, eccentricity, and length—and of the overall architecture of the vessel, including its tapering and curvature in the *x*, *y* and *z* coordinates. However, one needs to recognize the technical limitations and pitfalls of image acquisition modalities that account for error propagation prior to the implementation of 3D reconstruction algorithms. CCTA is the only truly volumetric image acquisition technique, but its spatial resolution is primarily determined by a voxel size that is limited to 0.4 mm^3^ in current clinical scanners [6]. On the contrary, nominal pixel size for current digital angiography systems ranges from 0.10 to 0.20 mm/pixel depending on detector matrix, table height, and magnification settings [10]. In the FFR2D study, average pixel size during calibration was 0.27 mm/pixel and minimum lumen diameter (MLD) was 1.76 ± 0.48 mm (e.g., 6–7 pixels) [9]. Consequently, a 1-pixel difference would account for 12–14% relative over- or underestimation of the MLD, which is the primary determinant of virtual FFR along with the microcirculatory resistance that governs blood flow rate across the stenosis. Studies have long shown a linear correlation with moderate agreement between 2D and 3D quantitative coronary angiography for characterization of the cross-sectional severity of coronary stenoses, but accuracy and diagnostic performance of, for example, the Murray law-based virtual QFR method, was almost identical when comparing 2D with 3D coronary angiographic analyses [3,11], supporting the notion that perhaps there is not much to be gained from 3D vessel reconstruction when considering the physical and technical limitations of current angiographic systems.

Of further interest, researchers have explored reduced-order CFD models to reduce complexity and computational burden associated with high-fidelity CFD simulations of 3D arterial models. Extensive sensitivity analyses have investigated the influence of model order reduction from 3D- to 2D- or even axisymmetric 1D-CFD models (the latter would be equivalent to the diameter function per unit of vessel length as produced by routine 2D-QCA analyses) in case of transient and steady (time average) flow under several variations of arterial geometry [12]. Not surprisingly, stenosis severity was the primary determinant of FFR uncertainty analysis, but a plain 2D model with time-averaged flow had a high diagnostic accuracy (0.99), while computational time was reduced by at least 5 orders of magnitude and the concurrent model error was well below clinical relevance [13]. Newman et al. had alternatively employed GPU processors to accelerate pseudo-transient 3D-CFD models in order to indirectly derive the coefficients *k*_1_ and *k*_2_ of the quadratic pressure drop function denoted in our FFR2D framework [9]: $\Delta p=k_{1}Q+k_{2}Q^{2}$.

Still, in spite of technical debates on 3D versus 2D approaches, and bias differences between analytical mathematical solutions versus CFD pipelines of variable order and resource consumption, the most influential parameter that affects virtual FFR prediction remains the simulated hyperemic microcirculatory capacity over the baseline conditions. The ratio of the latter refers to the well-known coronary flow reserve (CFR) that is measured with great variance with an invasive Doppler wire or thermodilution techniques during the pharmacological induction of hyperemia [14]. In-depth sensitivity simulations have identified that the factor by which the microvascular resistance is reduced during hyperemia is the leading governor of the corresponding relative increase of blood flow *Q* (also known as CFR), which in turn defines the magnitude of the measured or computed pressure drop in the abovementioned quadratic function [12].

Most established software solutions employ fixed values of microvascular resistance, blood flow velocity, or some other generic boundary condition to simulate hyperemic flow conditions. For example, the VirtuHeart approach by the team of Morris et al. assigns population averaged baseline and hyperemic microvascular resistances into a standard Windkessel model in order to compute vFFR [5]. On the contrary, a single-view angiographic approach of μQFR by Tu et al. assigns a hyperemic velocity value based on a previously fitted regression model in order to simulate hyperemia [2,3]. However, blood velocity may vary with acceleration across stenoses, whereas its baseline vessel-averaged value will be broadly stable with varying degrees of stenoses because of the coronary autoregulation [15]. On the contrary, in the FFR2D method, we opted for the use of blood (volumetric) flow rates that are largely stable across the interrogated vessel because of the conservation of mass, while ignoring losses from small side branches. Considering a well-established non-linear relationship between basal and hyperemic stenosis resistance and increasing diameter stenosis [15], we employed the TIMI Frame Count method [16] in order to, first, derive a patient-specific baseline value of coronary flow rate and, second, to simulate a geometry-specific value of CFR for the purpose of computing virtual FFR.

In summary, the authors believe that the proposed FFR2D analytical solution for computing virtual FFR from routine 2D angiographic images combines several inherent advantages and ease of use, while its proprietary anatomy-specific simulation of hyperemic flow helps achieve high accuracy and precision compared to wire-measured FFR.

## References

[1] Taylor D.J., Newman T., Gunn J., Morris P.D. (2025). Single View Techniques for Modelling Coronary Pressures Losses. Comment on Tsigkas et al. Rapid and Precise Computation of Fractional Flow Reserve from Routine Two-Dimensional Coronary Angiograms Based on Fluid Mechanics: The Pilot FFR2D Study. *J. Clin. Med.* 2024, *13*, 3831. J. Clin. Med..

[2] Tu S., Westra J., Yang J., von Birgelen C., Ferrara A., Pellicano M., Nef H., Tebaldi M., Murasato Y., Lansky A. (2016). Diagnostic Accuracy of Fast Computational Approaches to Derive Fractional Flow Reserve From Diagnostic Coronary Angiography: The International Multicenter FAVOR Pilot Study. JACC. Cardiovasc. Interv..

[3] Tu S., Ding D., Chang Y., Li C., Wijns W., Xu B. (2021). Diagnostic accuracy of quantitative flow ratio for assessment of coronary stenosis significance from a single angiographic view: A novel method based on bifurcation fractal law. Catheter. Cardiovasc. Interv..

[4] Newman T., Borker R., Aubiniere-Robb L., Hendrickson J., Choudhury D., Halliday I., Fenner J., Narracott A., Hose D.R., Gosling R. (2023). Rapid virtual fractional flow reserve using 3D computational fluid dynamics. Eur. Heart J. Digit. Health.

[5] Morris P.D., Silva Soto D.A., Feher J.F.A., Rafiroiu D., Lungu A., Varma S., Lawford P.V., Hose D.R., Gunn J.P. (2017). Fast Virtual Fractional Flow Reserve Based Upon Steady-State Computational Fluid Dynamics Analysis: Results From the VIRTU-Fast Study. JACC Basic Transl. Sci..

[6] Dell’Aversana S., Ascione R., Vitale R.A., Cavaliere F., Porcaro P., Basile L., Napolitano G., Boccalatte M., Sibilio G., Esposito G. (2023). CT Coronary Angiography: Technical Approach and Atherosclerotic Plaque Characterization. J. Clin. Med..

[7] Cortes C., Carrasco-Moraleja M., Aparisi A., Rodriguez-Gabella T., Campo A., Gutierrez H., Julca F., Gomez I., San Roman J.A., Amat-Santos I.J. (2021). Quantitative flow ratio-Meta-analysis and systematic review. Catheter. Cardiovasc. Interv..

[8] Fearon W.F., Achenbach S., Engstrom T., Assali A., Shlofmitz R., Jeremias A., Fournier S., Kirtane A.J., Kornowski R., Greenberg G. (2019). Accuracy of Fractional Flow Reserve Derived From Coronary Angiography. Circulation.

[9] Tsigkas G.G., Bourantas G.C., Moulias A., Karamasis G.V., Bekiris F.V., Davlouros P., Katsanos K. (2024). Rapid and Precise Computation of Fractional Flow Reserve from Routine Two-Dimensional Coronary Angiograms Based on Fluid Mechanics: The Pilot FFR2D Study. J. Clin. Med..

[10] Keane D., Haase J., Slager C.J., Montauban van Swijndregt E., Lehmann K.G., Ozaki Y., di Mario C., Kirkeeide R., Serruys P.W. (1995). Comparative validation of quantitative coronary angiography systems. Results and implications from a multicenter study using a standardized approach. Circulation.

[11] Ding D., Tu S., Chang Y., Li C., Xu B., Wijns W. (2022). Quantitative Flow Ratio Based on Murray Fractal Law: Accuracy of Single Versus Two Angiographic Views. J. Soc. Cardiovasc. Angiogr. Interv..

[12] Fossan F.E., Sturdy J., Muller L.O., Strand A., Braten A.T., Jorgensen A., Wiseth R., Hellevik L.R. (2018). Uncertainty Quantification and Sensitivity Analysis for Computational FFR Estimation in Stable Coronary Artery Disease. Cardiovasc. Eng. Technol..

[13] Gashi K., Bosboom E.M.H., van de Vosse F.N. (2019). The influence of model order reduction on the computed fractional flow reserve using parameterized coronary geometries. J. Biomech..

[14] Morris P.D., Curzen N., Gunn J.P. (2020). Angiography-Derived Fractional Flow Reserve: More or Less Physiology?. J. Am. Heart Assoc..

[15] Nijjer S.S., de Waard G.A., Sen S., van de Hoef T.P., Petraco R., Echavarria-Pinto M., van Lavieren M.A., Meuwissen M., Danad I., Knaapen P. (2016). Coronary pressure and flow relationships in humans: Phasic analysis of normal and pathological vessels and the implications for stenosis assessment: A report from the Iberian-Dutch-English (IDEAL) collaborators. Eur. Heart J..

[16] Gibson C.M., Cannon C.P., Daley W.L., Dodge J.T., Alexander B., Marble S.J., McCabe C.H., Raymond L., Fortin T., Poole W.K. (1996). TIMI frame count: A quantitative method of assessing coronary artery flow. Circulation.

